# Cesarean Section Complications Followed by Bladder Cystotomy and Gross Hematuria Due to Unknown Dense Scar Tissue

**DOI:** 10.7759/cureus.11902

**Published:** 2020-12-04

**Authors:** Nayda Parisio-Poldiak, Emma Morel, Christie Hua, Sean L Gibbs, David Billue

**Affiliations:** 1 Graduate Medical Education, Grand Strand Medical Center, HCA Healthcare, Myrtle Beach, USA; 2 Surgery, Edward Via College of Osteopathic Medicine-Carolinas, Myrtle Beach, USA; 3 Family Medicine, Grand Strand Medical Center, HCA Healthcare, Myrtle Beach, USA; 4 Obstetrics and Gynecology, OB Hospitalists Group, Myrtle Beach, USA

**Keywords:** hematuria, scar tissue, cesarean, bladder reconstruction, scar dehiscence

## Abstract

Adhesions formed from previous Cesarean section (C-section) are a significant risk factor for bladder injury. We present a case of a 43-year-old pregnant woman who underwent a C-section and experienced severe complications due to adhesions and incisional dehiscence from a previous Cesarean delivery 11 years earlier. Several surgical and non-surgical interventions as radiologic tests, cystotomy, blood transfusion, cystogram, and others were necessary to resolve the issues followed by the Cesarean delivery. It is important for clinicians caring for women undergoing both primary and subsequent Cesarean sections to consider and mitigate risk factors for adhesion development.

## Introduction

In the last several decades, Cesarean sections (C-sections) have become safer due to advances in perioperative pharmaceuticals, which decrease risks of infection and coagulopathy in the patient, along with enhancements in anesthesia and surgical techniques over the years. However, C-sections are still a surgical intervention employing abdominal and uterine incisions with risks of scarring and adhesions [1].

Women aged ≥40 undergoing Cesarean deliveries are more likely to have complications compared to women <40. Those issues include a 75% increase in intraoperative transfusion, 38% increase in postpartum transfusion, three times increase in bowel injury, 92% increase in abnormal placentation, 59% increase in the classical incision, and two times increase in Cesarean hysterectomy and maternal intensive care unit (ICU) admission [2]. In addition, pregnancy outcomes at very advanced maternal age have a higher rate of maternal complications such as gestational hypertension, preeclampsia, gestational diabetes, increased need for blood transfusion, and prolonged hospitalization [3].

Due to the increased use of Cesarean deliveries, the development of post-Cesarean adhesions is a much more common occurrence leading to a wide variety of complications. Adhesions formed from previous C-sections are a significant risk factor for bladder injury [4]. The incidence of adhesion development after primary Cesarean ranges from 46-65% and increase with each subsequent Cesarean section [5]. Repeat Cesarean deliveries can result in signiﬁcant complications due to adhesive disease caused by previous surgeries.

We present a case of a 43-year-old pregnant woman who underwent a Cesarean section and experienced severe complications due to dense scar tissue from a previous Cesarean delivery 11 years earlier. Several surgical and non-surgical interventions as radiologic tests, cystotomy, blood transfusion, cystogram, and others were necessary to resolve the issues followed the Cesarean delivery.

## Case presentation

**Figure 1:**
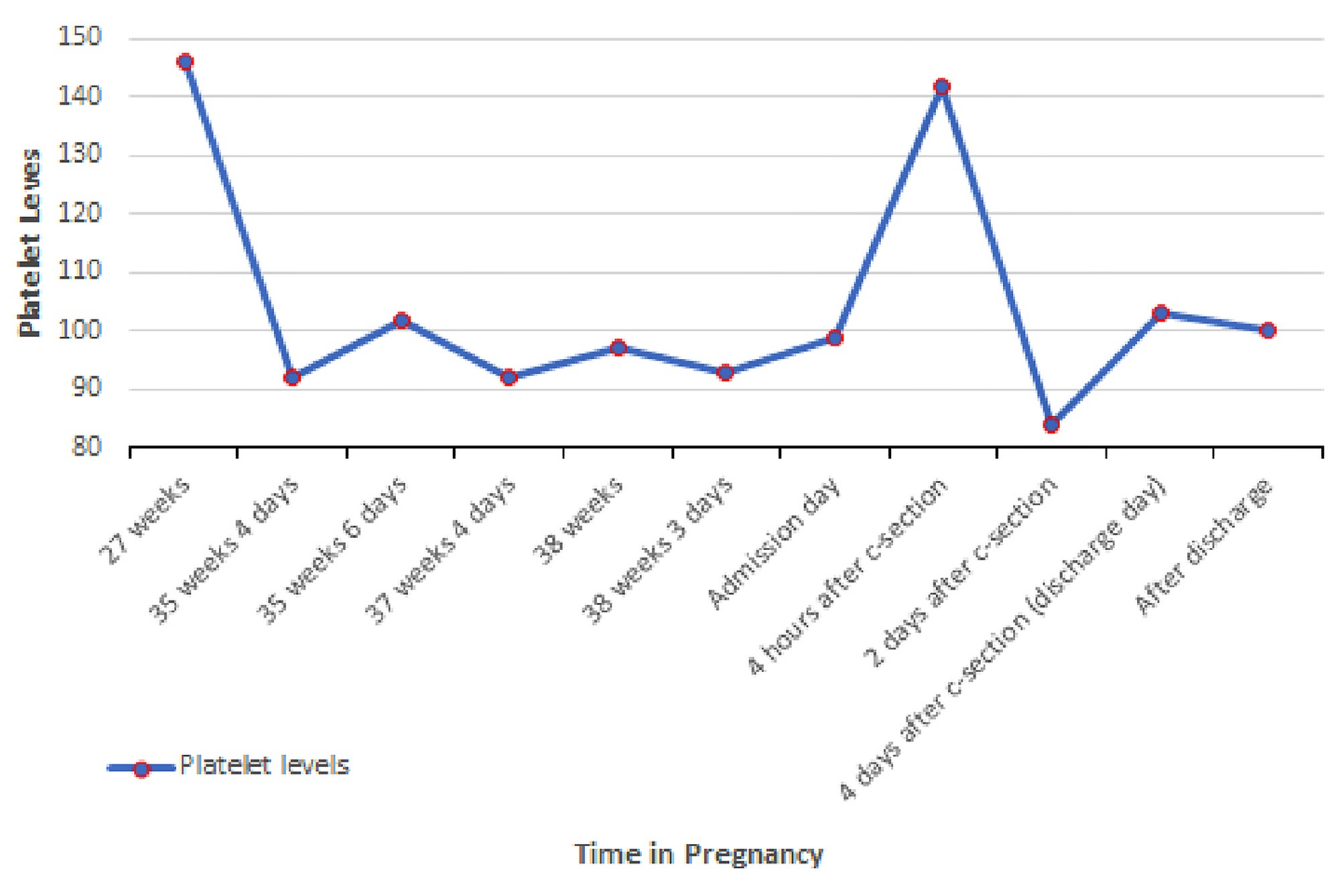
Platelets level during the pregnancy Normal range: 156-352x10^3^/mL

A 43-year-old pregnant woman G2P2 at 38 weeks with no significant past medical history presented to the primary care clinic two days before the scheduled elective Cesarean section with symptoms of labor including abdominal pain, contractions every 20 minutes, and normal fetus movement. On vaginal examination, the patient’s cervix was dilated to 3 cm, effacement at 80%, and located at** **-1 (minus one) station. The patient was sent to hospital labor and delivery (HLD) for monitoring. Overall, the patient’s prenatal course has been uneventful except for the development of gestational thrombocytopenia during the third trimester (Figure 1). On HLD, the patient had a reactive non-stress test with contractions every four to six minutes. A cervical exam revealed an increased cervical effacement. After discussion with the patient, the team elected to proceed with a repeat Cesarean section delivery due to labor. The patient did not desire a trial of labor. The patient was admitted and taken to the operating room that evening. The patient’s first pregnancy 11 years prior was unremarkable, and she had an uncomplicated elective Cesarean section.

Upon arrival in the operating room, the patient had stable vital signs with a heart rate of 65 beats/min, blood pressure (BP) of 143/80, and pulse oxygen saturation of 100%. Spinal anesthesia was performed in a sitting flexed-back position at the L3 to L4 interspace. Pfannenstiel's incision was made from excising the prior scar. The delivery was notable for significant scar tissue from the previous Cesarean section. The bladder peritoneum was attached very high on the uterus near the fundus. There was also the separation of the Cesarean section scar (uterine scar dehiscence) and significant thinning of the bladder peritoneum above the scar with a window present. The bladder was covering the opening, and its reflection was carefully dissected off the uterus. The uterine cavity was entered without an incision, and the baby was delivered thru the opening after the bladder peritoneum was separated from the uterus. Amniotic fluid was clear. After delivery of the infant, an incidental 4cm cystotomy in the dome of the bladder was noted. The trigone area, the urethra, and ureter openings were not involved in the laceration. The very thin uterine muscle was then carefully dissected from the scarred bladder wall. The obstetrician repaired the bladder in a two-layer fashion using 3.0 absorbable, synthetic suture with good results and no leakage evidence. Estimated blood loss was around 750 cc. There were no other problems during the delivery, and a healthy male infant was born with Apgar 8/9.

**Figure 2:**
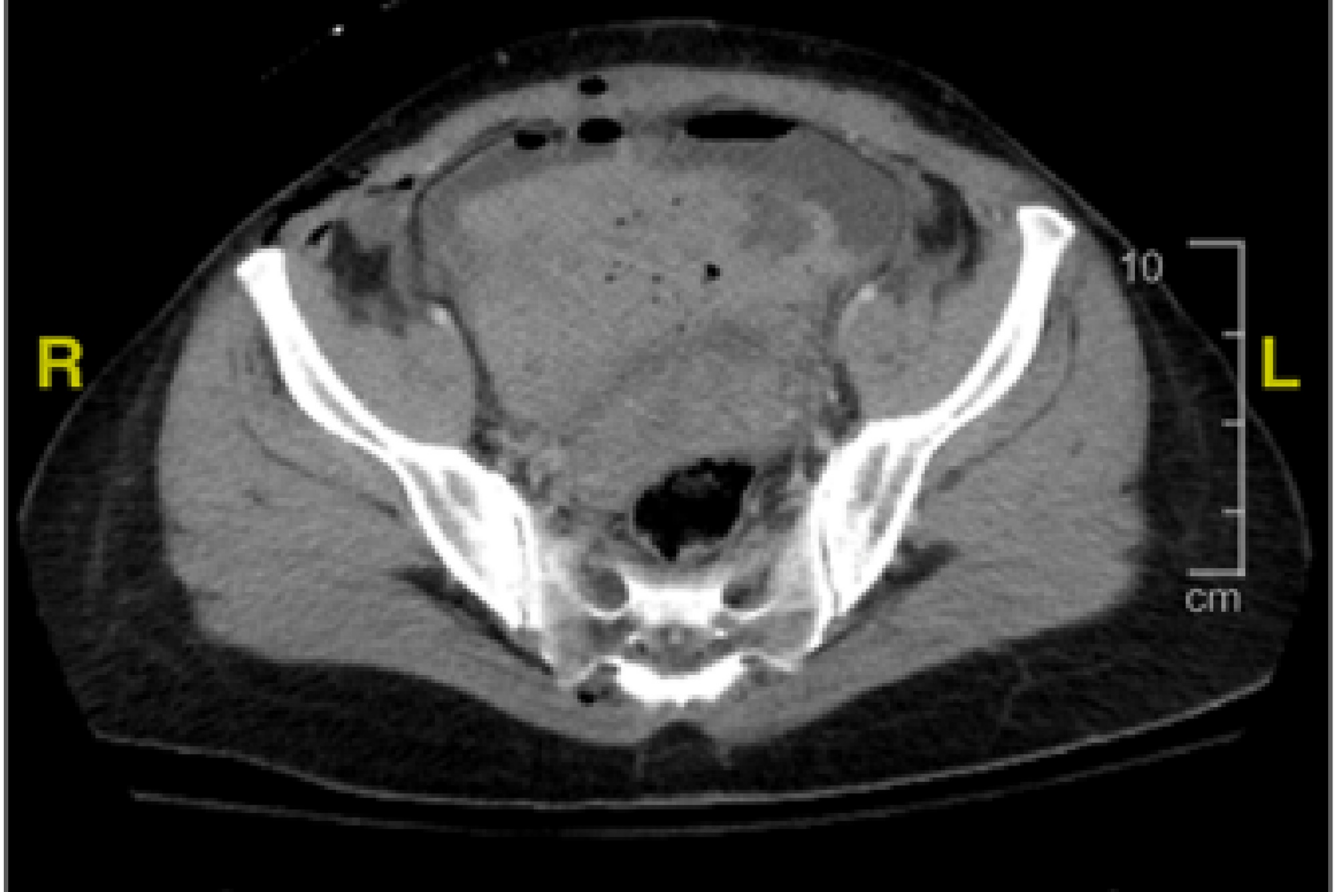
CT scan showing blood clot inside the bladder

**Figure 3:**
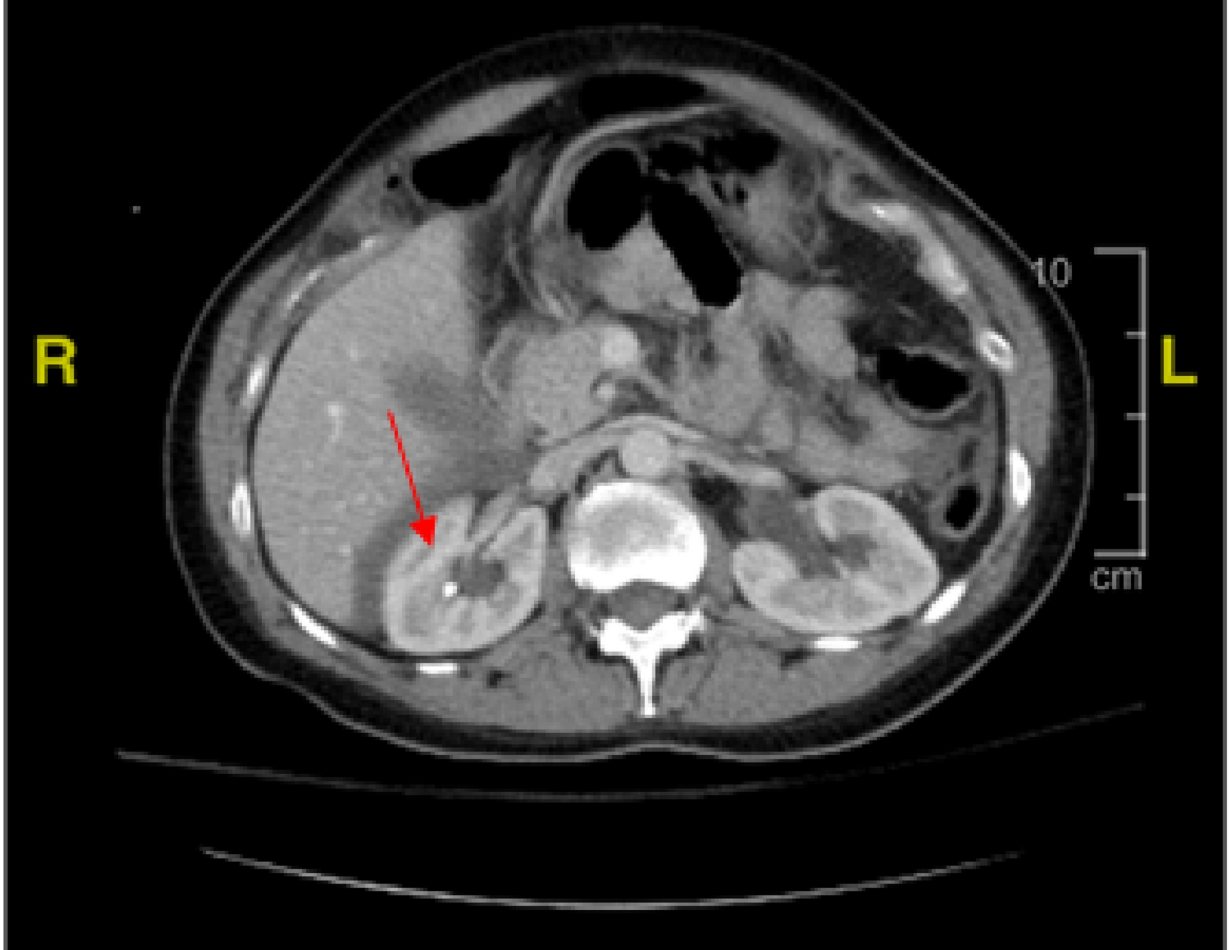
CT scan showing nephrolithiasis in the right kidney

Approximately two hours after surgery, she developed tachycardia and intermittent episodes of low blood pressure (Table 1). Hemoglobin was rechecked and was 9.9, which had decreased from 13 on admission (Table 2). It was elected to give her two units of packed red blood cells due to blood loss anemia to improve oxygen-carrying capacity. The patient continued to have bloody urine output following delivery. Urine output was adequate. The Foley catheter clotted off, and the urinary catheter had to be replaced. Post transfusion patient’s hemoglobin was 9. The patient's vital signs improved, and the pain was managed. She continued to have bloody urine output into the morning after delivery. CT scan showed a mild amount of fluid in the pelvic cavity and abdomen, with no sign of intra-abdominal bleeding or hematoma, and abnormal high-density material in the bladder lumen appeared to be a blood clot (Figure 2). The uterus was normal post-delivery. Urology was consulted, and a larger catheter was placed in the bladder to prevent the clot from obstructing the flow of urine output. The Foley catheter was changed from a 16-to 22-French Foley to a 24 F Couverlaire tip catheter. There continued to be intermittent problems with the catheter being blocked despite irrigation with saline. The patient reported an extreme right-sided pain radiating to the neck and shoulder area during the Foley cleaning procedures (pain level 8-10) that could be associated with the kidney stone (Figure 3).

**Table 1:** Vital signs during hospitalization Vital signs before and after the patient passed through C-section. Measurements: temperature in Fahrenheit, pulse in beats per minute, blood pressure in millimeters of mercury (mmHg), pulse oximetry in percentages.

Vital signs	Temperature	Pulse	Blood pressure	Pulse oximetry
Admission	98.6	81	141/94	-
C-section	97.2	65	143/80	100
2 hours later	-	80	87/50	99
3 hours later	-	84	68/40	94
4 hours later	-	104	111/60	99
6 hours later	99	113	79/49	98
16 hours later	99.1	83	144/78	98
24 hours later	99	75	123/68	98
48 hours later	100.3	93	127/70	95
72 hours later	99	88	124/70	99

**Table 2:** Hematology results Hours counted after the C-section. HgB - hemoglobin, reference range: 11.6 – 15.4 mg/dl; HcT - hematocrit, reference range: 34.9 – 44.1 %; WBC - white blood cells, reference range: 3.7 – 10.1 K/mm3, platelets reference range: 156-352 x103/mL

Hematology	HgB	HcT	WBC	Platelets
24 hours before admission	12.4	36.3	12.0	93
Admission	13.0	38.4	15.1	99
4 hours later	9.9	29.7	28.3	142
7 hours later (2 units - blood transfusion)	9.0	27.3	-	-
9 hours later	10.0	30.3	-	-
32 hours later (1 unit - blood transfusion)	8.6	23.9	12.0	84
40 hours later	8.6	25.0	15.0	-
7 days later	11.0	31.8	9.2	100

On the following day, the patient had another episode of weakness with symptomatic tachycardia. She was given the third unit of blood without complications. Post transfusion, her hemoglobin was 8.6 (Table 2). The patient was taken to the operating room (OR) by urology that day, and a cystoscopy, evacuation of clots, and cystogram were performed. A significantly organized and solid clot with a volume of ~500 cc was removed from the bladder under general anesthesia. A contrast-enhanced cystogram was completed showing no evidence of extravasation of contrast from the urinary bladder. At that time, the bladder was re-distended, and there was no leakage from the bladder. Subsequently, she improved quickly and had no further bleeding episodes. The pain was controlled, and urine became clear, with no further catheter problems. The patient was discharged home on the fifth day and was instructed to follow up with urology in approximately 10 days for a catheter check. She was given a prescription for pain medications, continued antibiotics for urinary tract infection prophylaxis, vitamins, and iron. Ten days after discharge, during the follow-up visit with the urologist, a bladder scan showed moderate urinary retention (post-void residual urine = 381 cc) upon removal of the catheter. The patient was followed at regular intervals until resolution of retention, with instructions to return if symptoms developed. Later outpatient follow-up revealed complete resolution at 18 months.

## Discussion

In the last few decades in the U.S., there has been a dramatic increase in maternal age at childbearing due to a number of factors, including cultural and socioeconomic practices [6]. This trend has perpetuated globally despite well-established risks of pregnancy in advanced maternal age, especially in women >40 years old [7]. As the population of older women pursuing pregnancy grows, the data demonstrates strengthening and clear relationships between advanced maternal age and intra- and peripartum complications involving both the mother and fetus. A study of over 60,000 mothers, 2,436 who were over age 40, found a higher rate of transfusions of packed red blood cells (PRBCs), maternal ICU admission, bowel injury, placenta accreta, and use of classical uterine incision compared to the control group [2]. A multivariate analysis study found maternal age greater than 40 to be an independent risk factor for preterm delivery, Cesarean section, abnormal fetal presentation, and fetal periventricular leukomalacia [8].

Assessing blood counts in pregnancy poses specific challenges that can make the decision to transfuse the patient much less straightforward than a non-pregnant patient. During pregnancy, there is a disproportionate increase in plasma volume (50%) compared to red blood cell mass (20-30%), creating dilutional anemia, which is considered normal physiology in pregnant females. In addition, the pregnant patient becomes hypercoagulable with increased production of fibrinogen and factors VII, VIII, and IX compared to anticoagulants protein A, protein C, and antithrombin III [9]. In a non-pregnant patient, blood transfusion is typically not warranted with a hemoglobin >7 g/dL; however, in the pregnant or postpartum patient, additional factors such as clinical presentation, maternal vital signs, and the presence or absence of fetal distress should be taken into consideration before the decision to transfuse. In our case, the patient’s hemoglobin fell from 13 g/dL to 9 g/dL with a known source of bleeding (bladder injury), which necessitated transfusion. 

Progressive thrombocytopenia is also considered a normal physiologic change of pregnancy, beginning in the second trimester, due to the dilutional effect of increased plasma volume. Gestational thrombocytopenia is defined as a platelet count below 150 x 109/L and occurs in up to 11.6% of pregnancies [10]. The spectrum of thrombocytopenia in pregnancy culminates with hemolysis syndrome, elevated liver enzymes, and low platelets (HELLP), which occurs in 9.5 per 1,000 deliveries [11]. In our case, the patient experienced gestational thrombocytopenia beginning at 35 weeks gestation, which was followed by a further drop in platelets in the post-partum period as well as after receiving and a PRBC transfusion with nidus at 84 x 109/L at 36 hours after Cesarean section.

Current Royal College of Obstetricians & Gynaecologists Green-top Guidelines suggest the following goals of transfusion therapy in the obstetric patient: hemoglobin >8 g/dL, platelet count >75 x 109/L, prothrombin time <1.5 x mean control, activated prothrombin time (PT) <1.5 x mean control, and fibrinogen >1.0 g/L [10]. Again, it should be emphasized to consider the entire clinical picture when making the decision to transfuse, as in our case, the patient did not perfectly fit the traditional criteria for transfusion; however, the transfusions were imperative to the care and successful recovery of the patient.

Adhesions and scar tissue formation are physiologic responses to injury and tissue disruption. They are composed of coagulation factors and inflammatory cells weaved within a fibrinous matrix and begin to form immediately after an insult due to an imbalance between fibrin deposition and fibrinolysis [12]. Lyell reports an incidence of 46-65% for adhesions following primary Cesarean delivery and even greater rates amongst women who underwent subsequent Cesarean deliveries [5]. Furthermore, the density of adhesions is found to be increasingly greater with increasing numbers of Cesarean deliveries [13]. In our case, there was a significant amount of scar tissue formation, which created an attachment between the uterus and bladder, likely contributing to the complication of a cystotomy.

Bladder injury is a rare complication of Cesarean deliveries and has been associated with adhesion formation. One study assessing 14,757 Cesarean sections at a large academic center over seven years found the incidence of bladder injury in repeat Cesarean sections to be 0.28% (42/14,757 women), with women having repeat Cesarean deliveries having nearly four times increased incidence of bladder injury [14]. Employing a careful, sharp dissection technique may reduce dense scar tissue formation, thus reducing the chances of bladder injury in subsequent gynecological manipulation. Interestingly, the rate of cystotomy in women undergoing laparoscopic hysterectomy was found to be 21.1% in women who had at least three Cesarean deliveries compared to 1.2% in women without previous Cesarean delivery (n=572, adjusted odds ratio 18.4, 95% CI: 5.2 - 66) [15].

Bladder injuries most commonly occur during the creation of the bladder flap (43%), followed by surgical entrance into the peritoneal cavity (33%) and finally during uterine incision (24%). The majority of bladder injuries are discovered intraoperatively and undergo immediate repair with an excellent return to function post-operatively [14]. Failure to recognize bladder injury increases the risk of vesicovaginal, vesicouterine, or uterovaginal fistulae. Intraoperative findings suggestive of bladder injury include urine extravasation, visualization of the Foley bulb, gross hematuria, and visible violation of bladder mucosa [16]. Post-operative signs and symptoms of missed cystotomy include hematuria, oliguria, lower abdominal pain, ileus, ascites, peritonitis, sepsis, and elevated blood urea nitrogen and creatinine levels [17]. In our case, the bladder defect was visualized during the initial operative period with the successful two-layer repair.

Bleeding and hematoma formation are common and well-documented complications of any surgical intervention. Upon a thorough literature review, no cases to our knowledge of significant bladder hematoma complications after Cesarean section bladder repair. The most common cause of intraluminal urinary bladder hematoma is trauma, which is managed with cystography with hematoma evacuation under CT or X-ray guidance [18]. In our case, the patient presented with anemia not responsive to multiple transfusions and was found to have a large clot burden (500cc) in the bladder on cystographic inspection. A Foley catheter was placed post-operatively, per American Urological Association (AUA) guidelines, which recommend 7-14 days of indwelling Foley catheter following bladder injury [19]. However, the bladder was inadequately decompressed due to the large hematoma. Following the surgical evacuation of the hematoma, the patient recovered without further complication. 

## Conclusions

It is important for clinicians caring for women undergoing both primary and subsequent Cesarean sections to consider and mitigate risk factors for scar tissue and adhesion development. Practitioners should maintain a high index of suspicion of blood dyscrasias and injury to surrounding organs and tissues during the pre and post-natal periods. Failure to properly diagnose and promptly manage maternal complications may lead to adverse outcomes, including bladder rupture, massive hemorrhage, and other severe complications. Recognition of the aforementioned problems leads to improved outcomes and the ultimate safety of both patient and neonate.
